# Supplementary material for: Carbene Addition Isomers of C70 formed in the Flame of Low-Pressure Combustion
Source: Nanomaterials (Basel). 2022 Sep 6;12(18):3087. doi: 10.3390/nano12183087 (PMC9503469; doi:10.3390/nano12183087)
Supplement: Supplementary file 1 [file nanomaterials-12-03087-s001.zip › nanomaterials-1890964-supplementary.pdf]

Supplementary Materials

Carbene Addition Isomers of C_{70} formed in the Flame of Low-Pressure Combustion

Fang-Fang Xie, Zuo-Chang Chen *, You-Hui Wu, Han-Rui Tian, Shun-Liu Deng *, Su-Yuan Xie and Lan-Sun Zheng

State Key Lab for Physical Chemistry of Solid Surfaces, Collaborative Innovation Center of Chemistry for Energy Materials, Department of Chemistry, College of Chemistry and Chemical Engineering, Xiamen University, Xiamen 361005, China

* Correspondence: zcchem@126.com (Z.-C.C.); sldeng@xmu.edu.cn (S.-L.D.)

1. Separation of $C_{71}H_2$-I, $C_{71}H_2$-II and $C_{71}H_2$-III

The carbon soot (about 500g) synthesized from low combustion was dissolved in toluene and the soluble products were extracted by ultrasound. The extraction rate of soluble extracts can only be roughly estimated to be 2.5% (about 12.5g) because the soluble products such as fullerenes and PAHs could not be extracted completely, even many times of extraction, from carbon soot. Since the products were all in solution during HPLC separation, the peak area normalization method was used to estimate the product yields. As shown in the first stage of HPLC separation (Figure S1a/S2a), the total soluble products typically contain empty fullerene cages (about 40%), hydrogenated PAHs (about 40%), hydrofullerenes and hydrofullerene derivatives (about 20%). The yield of C_{60} was quantitatively analyzed by the peak area method, that is, the yield of C_{60} was obtained by dividing the peak area of C_{60} by the total peak area in Figure S1a. The yields of C_{70}, $C_{71}H_2$-I, $C_{71}H_2$-II and $C_{71}H_2$-III were calculated in the same way as that of C_{60}. Further HPLC separation processes reveal that the contents of C_{60}, C_{70}, $C_{71}H_2$-I, $C_{71}H_2$-II and $C_{71}H_2$-III are approximately 20% (about 2.5 g), 10% (about 1.25 g), 0.00028% (about 0.035 mg), 0.00144% (about 0.18 mg), 0.0068% (about 0.85 mg) of the total soluble products, respectively. Among them, the yields of $C_{71}H_2$-I, $C_{71}H_2$-II and $C_{71}H_2$-III in soluble extracts at each separation stage are shown in the Table S1 below.

Table S1. The yields of $C_{71}H_2$-I, $C_{71}H_2$-II and $C_{71}H_2$-III in soluble extracts at each separation stage.

	**$C_{71}H_2$-I**	**$C_{71}H_2$-II**	**$C_{71}H_2$-III**
First stage yield	0.5%	0.5%	0.5%
Second stage yield	5.4%	5.4%	5.4%
Third stage yield	7%	7%	38%
Fourth stage yield	15%	76%	67%
Total yield (%)	0.00028%	0.00144%	0.0068%
Total yield (mg)	0.035 mg	0.18 mg	0.85 mg

$C_{71}H_2$-I and $C_{71}H_2$-II. The procedures for the isolation and purification of $C_{71}H_2$-I/II include four stages of HPLC run, of which the last run was carried out in a recycling mode. In each stage, the samples containing $C_{71}H_2$-I/II were collected and then confirmed by mass spectrometry. First, the crude toluene extracts of carbon soot were roughly separated into several components by a Buckyprep column (i.d. 20 × 250 mm), where the main component containing $C_{71}H_2$-I/II from 15.5 to 18.8 min was collected (Figure S1a). Second, the collected samples were isolated using a 5PBB column, and then the $C_{71}H_2$-I/II-containing component was collected from 16.9 to 19.0 min (Figure S1b). Third, the isolation was performed by a Buckyprep column (i.d. 10 × 250 mm) and the component containing $C_{71}H_2$-I/II from 15.0 to 16.0 min was collected (Figure S1c). Finally, the last stage of purification was conducted on a 5NPE column in a recycling mode, and the purified $C_{71}H_2$-I (shown as red) and $C_{71}H_2$-II (shown as green) were obtained eventually (Figure S1d). The

conditions of the separation process were indicated in the upper right corner of each chromatogram.

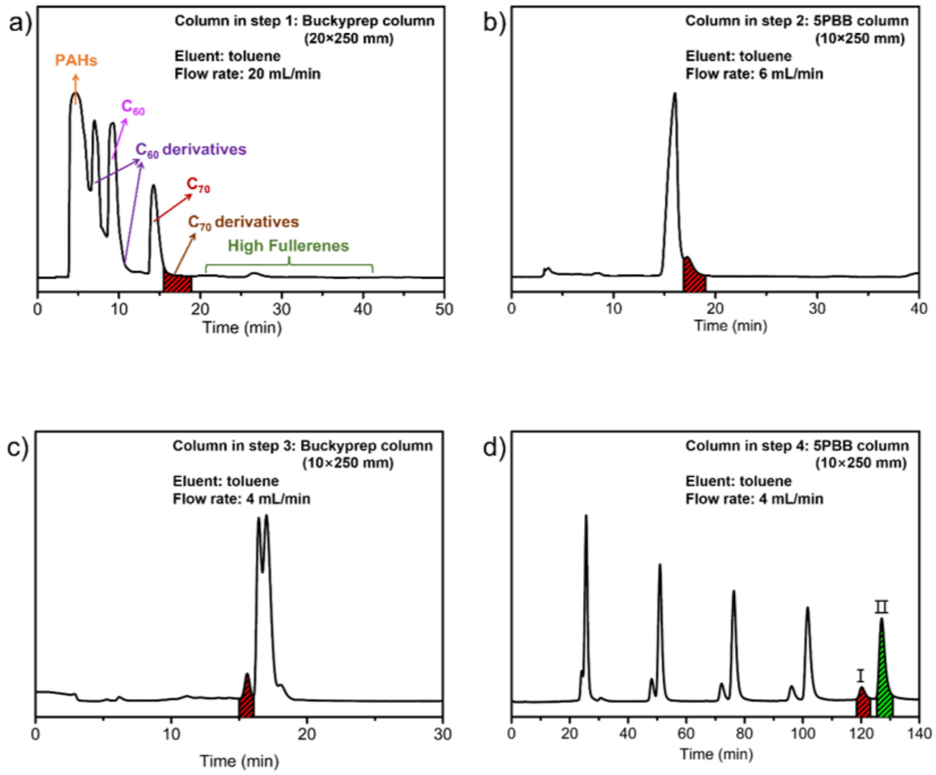

Figure S1. HPLC chromatograms for the separation of $C_{71}H_2$-I and $C_{71}H_2$-II. (The shaded areas correspond to the collected components containing $C_{71}H_2$-I/II). (**a**), (**b**), (**c**) and (**d**) correspond to the HPLC separation chromatograms of $C_{71}H_2$-I and $C_{71}H_2$-II in the first, second, third and fourth stage, respectively.

$C_{71}H_2$-III. Separation and purification of $C_{71}H_2$-III were conducted under the same HPLC conditions as $C_{71}H_2$-I/II, which also include four stages of HPLC run, of which the last run was carried out in recycling mode. The first and second stages (Figure S2a, S2b) of the $C_{71}H_2$-III isolation processes were identical to those of $C_{71}H_2$-I/II as mentioned in Figure S1. In the third stage, the collected samples from the second stage were divided into several fractions with a Buckyprep column (i.d. 10 × 250 mm) and the fraction containing $C_{71}H_2$-III from 16.7 to 17.8 min was collected (Figure S2c). Finally, the sample of $C_{71}H_2$-III was purified in a recycling mode by a 5PBB column to afford the $C_{71}H_2$-III sample with high purity (Figure S2d).

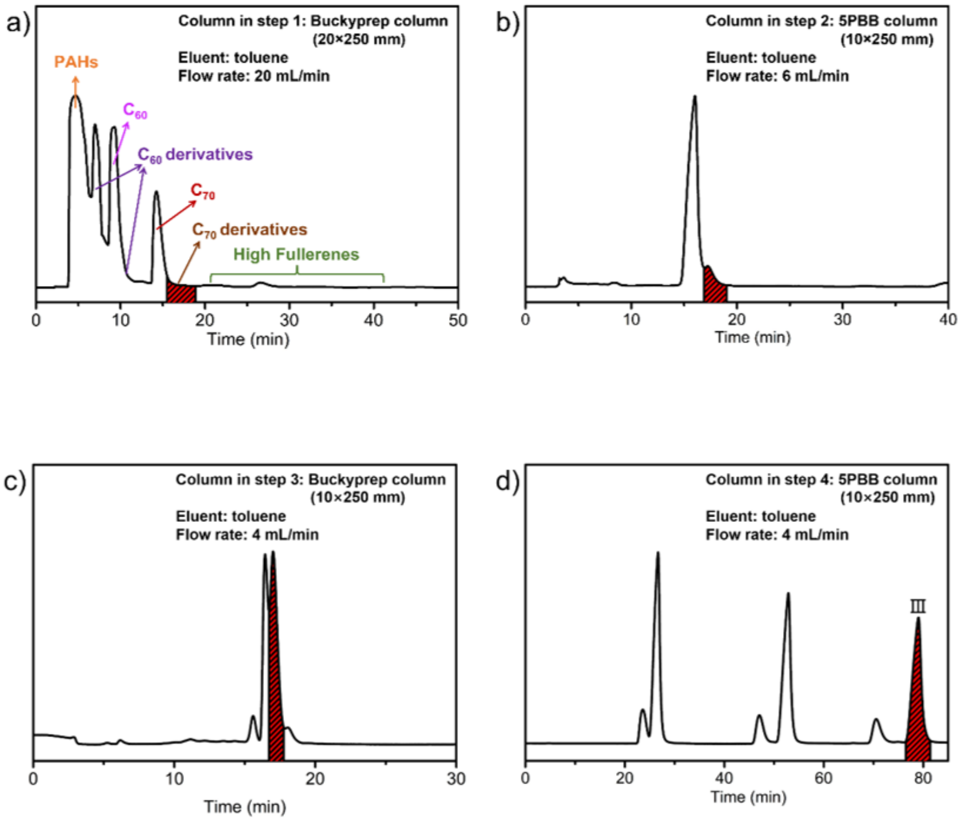

Figure S2. HPLC chromatograms for the separation of $C_{71}H_2$-III. (The shaded areas correspond to the collected components containing $C_{71}H_2$-III). (**a**), (**b**), (**c**) and (**d**) correspond to the HPLC separation chromatograms of $C_{71}H_2$-III in the first, second, third and fourth stage, respectively.

2. APCI-MS spectra of $C_{71}H_2$-I, $C_{71}H_2$-II and $C_{71}H_2$-III

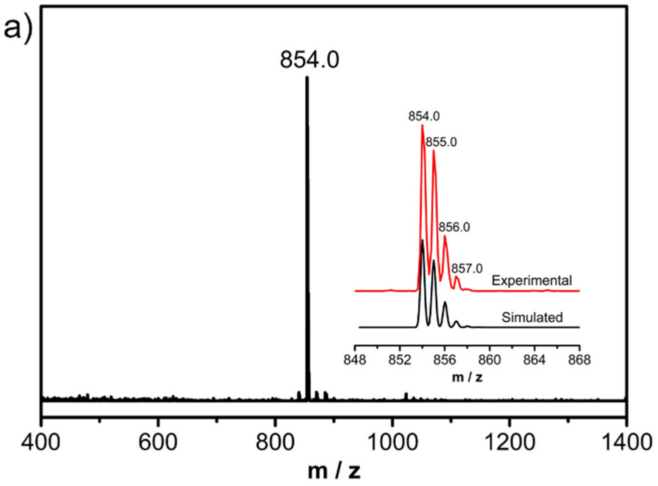

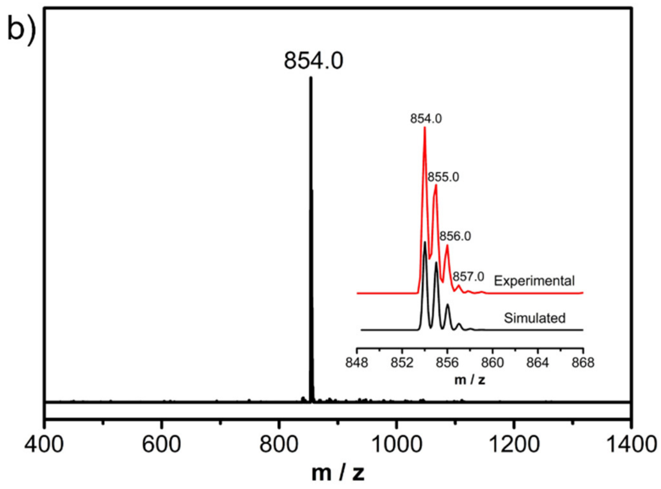

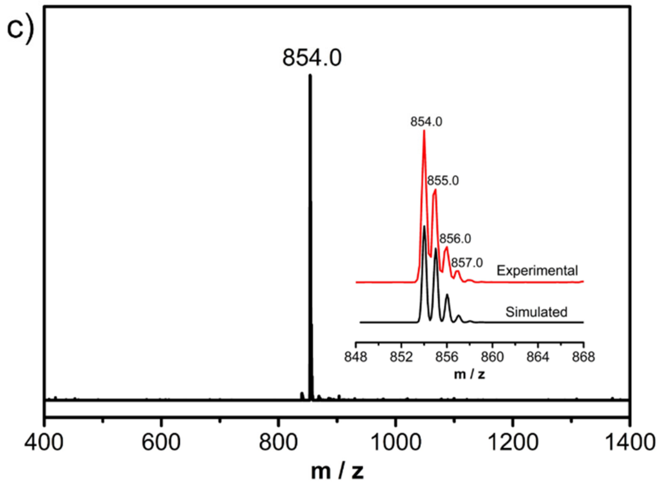

Figure S3. APCI-MS spectra of $C_{71}H_2$-I (**a**), $C_{71}H_2$-II (**b**) and $C_{71}H_2$-III (**c**) in toluene. (The insets show the experimental and simulated isotopic distributions).

3. MS/MS spectra of $C_{71}H_2$-III, $C_{71}H_2$-II and $C_{71}H_2$-III

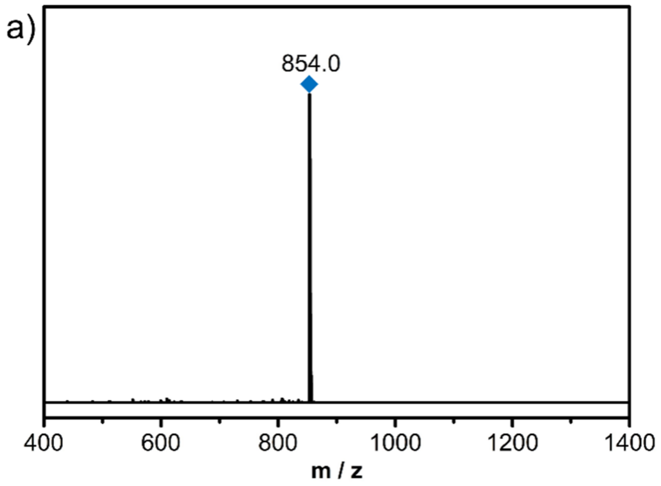

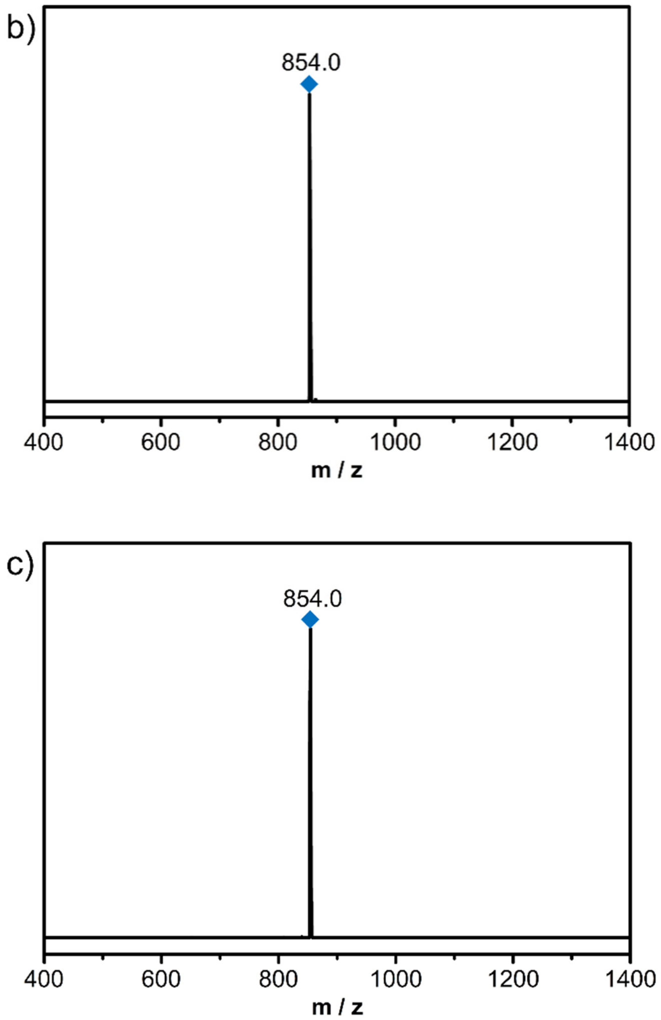

Figure S4. MS-MS spectra of $C_{71}H_2$-I (**a**), $C_{71}H_2$-II (**b**) and $C_{71}H_2$-III (**c**) in toluene.

4. Crystallographic information

The crystallographic data for $C_{71}H_2$-I, $C_{71}H_2$-II and $C_{71}H_2$-III have been deposited at the Cambridge Crystallographic Data Centre (CCDC) under the deposition number CCDC 1856262, 1856263 and 1827408 respectively, which can be obtained free of charge from www.ccdc.cam.ac.uk/data request/cif (accessed on 10 August 2022).

The units containing DPC molecules and three $C_{71}H_2$ isomers molecules are respectively shown in Figure S5, where the toluene molecules are omitted for clarity. In addition, crystallographic data and other details of 2DPC{$C_{71}H_2$-I}, 2DPC{$C_{71}H_2$-II} and 2DPC{$C_{71}H_2$-III} are listed in Table S1.

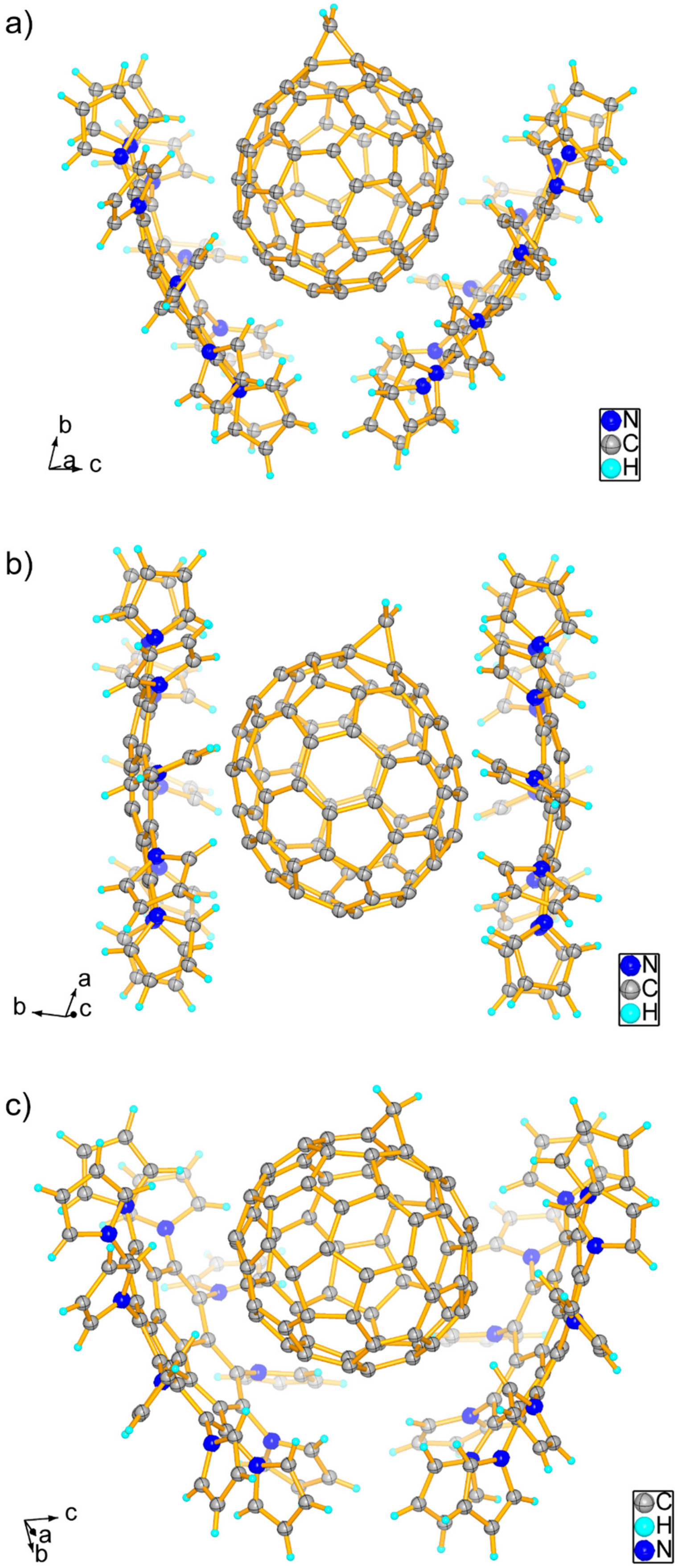
a)
b
a
c
N
C
H
b)
a
b
c
N
C
H
c)
c
a
b
C
H
N

Figure S5. $C_{71}H_2$-I (**a**), $C_{71}H_2$-II (**b**) and $C_{71}H_2$-III (**c**) molecules and two co-crystallized DPC molecules in the unit. (C gray, H cyan, N navy blue).

Table S2. Crystallographic data for $C_{71}H_2$-I, $C_{71}H_2$-II and $C_{71}H_2$-III.

	**2DPC{$C_{71}H_2$-I}**	**2DPC{$C_{71}H_2$-II}**	**2DPC{$C_{71}H_2$-III}**
**Empirical formula**	**$C_{212}H_{106}N_{20}$**	**$C_{219}H_{114}N_{20}$**	**$C_{205}H_{98}N_{20}$**
**formula weight**	2933.16	3025.30	2841.03
**Temperature/K**	100K	100K	150K
**crystal system**	triclinic	triclinic	triclinic
**space group**	P-1	P-1	P-1
***a*/Å**	14.8351(6)	14.4222(5)	14.7974(4)
***b*/Å**	17.4182(8)	17.4610(5)	17.0624(4)
***c*/Å**	30.0997(7)	18.1172(7)	30.7179(7)
**α/deg**	78.223(3)	113.558(3)	78.188(2)
**β/deg**	78.217(3)	106.695(3)	78.421(2)
**γ/deg**	69.831(4)	98.916(2)	71.184(2)
**Volume/Å^3**	7071.0(5)	3812.0(2)	7109.4(3)
Z	2	1	2
**ρ_{calc}/g·cm^{-3}**	1.378	1.318	1.327
**μ/mm^{-1}**	0.639	0.609	0.618
***F* (000)**	3036	1568	2936
**Crystal size/mm^3**	0.2×0.15×0.1	0.2×0.15×0.1	0.3×0.2×0.2
**Radiation**		Cu Kα (λ = 1.54184)	
**2θ range/°**	6.896 to 125.6	7.41 to 125.588	6.634 to 131.628
**parameters**	2273	1398	2274
**Index ranges**	$-17 \le h \le 16$<br>$-20 \le k \le 17$<br>$-34 \le l \le 25$	$-10 \le h \le 16$<br>$-20 \le k \le 18$<br>$-20 \le l \le 20$	$-17 \le h \le 17$<br>$-20 \le k \le 20$<br>$-36 \le l \le 36$
**Reflections collected**	44880	24034	112046
**Independent reflections**	22587	12205	24302
**Goodness-of-fit on F^2**	1.015	1.037	1.039
**Final R indexes [I>=2σ (I)]**	R_1 = 0.0815<br>wR_2 = 0.2047	R_1 = 0.0547<br>wR_2 = 0.1396	R_1 = 0.1147<br>wR_2 = 0.2968
**Final R indexes [all data]**	R_1 = 0.1137<br>wR_2 = 0.2383	R_1 = 0.0633<br>wR_2 = 0.1485	R_1 = 0.1338<br>wR_2 = 0.3166

$R1 = \Sigma| |Fo|-|Fc| |/\Sigma|Fo|$, $wR2 = [\Sigma w(Fo^2-Fc^2)^2]/\Sigma w(Fo^2)^2]^{1/2}$

5. UV-Vis spectra of $C_{71}H_2$-I, $C_{71}H_2$-II and $C_{71}H_2$-III

The UV-Vis data of $C_{71}H_2$-I, $C_{71}H_2$-II and $C_{71}H_2$-III were recorded on a Shimadzu UV-2550 UV-Vis spectrophotometer in toluene. As shown in Figure 3, the UV-Vis spectra of $C_{71}H_2$-I, $C_{71}H_2$-II and $C_{71}H_2$-III were put together for comparison, which show broad absorptions at 374, 445, 540, 590, 640, 688 nm for $C_{71}H_2$-I; 372, 403, 463, 540, 608, 666 nm for $C_{71}H_2$-II and 379, 475, 513, 550, 595, 612, 638 nm for $C_{71}H_2$-III, respectively. There is no absorption in the longer wavelength region.

6. Theoretical calculations

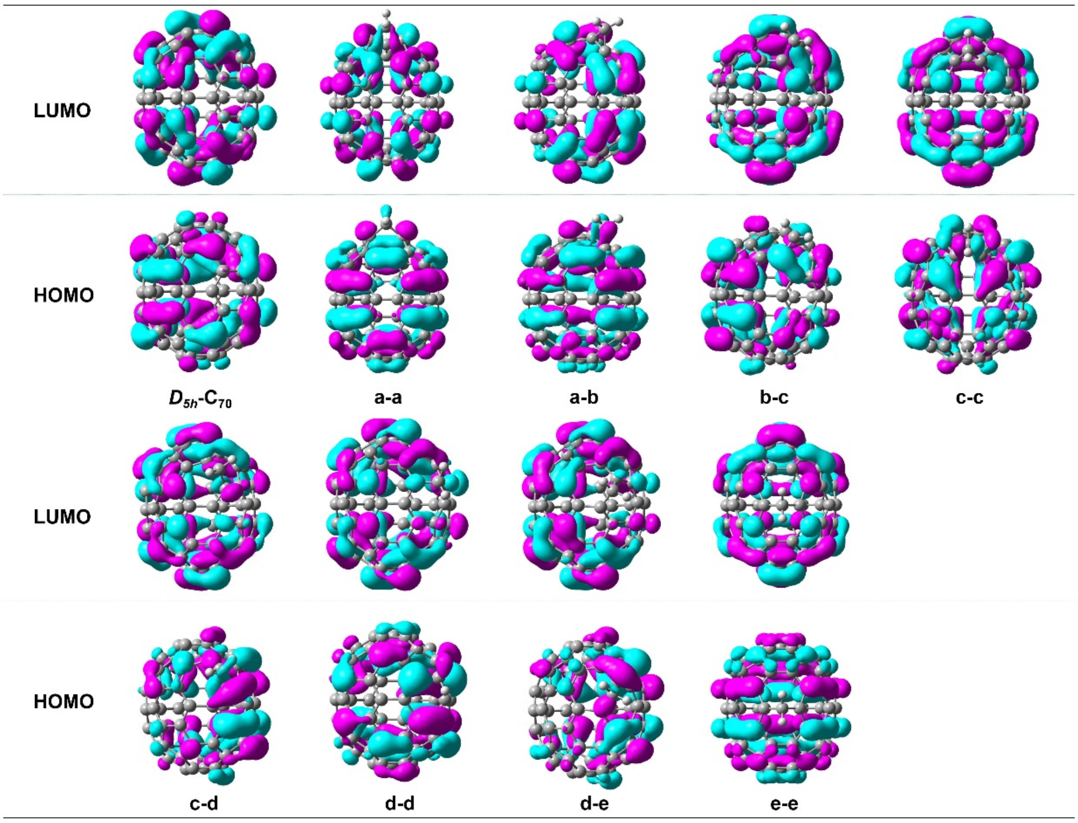

Figure S6. The highest occupied molecular orbitals and lowest unoccupied molecular orbitals of $C_{71}H_2$ isomers and D_{5h}-C_{70} at B3LYP/6-31G(d,p) level.

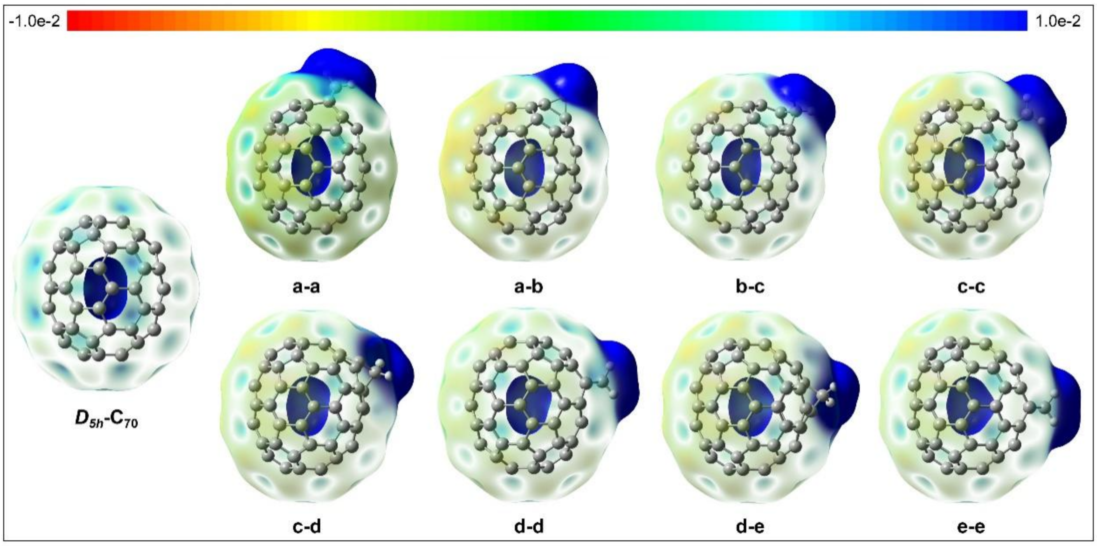

Figure S7. The electrostatic potential surfaces of $C_{71}H_2$ isomers and D_{5h}-C_{70} at B3LYP/6-31G(d,p) level.

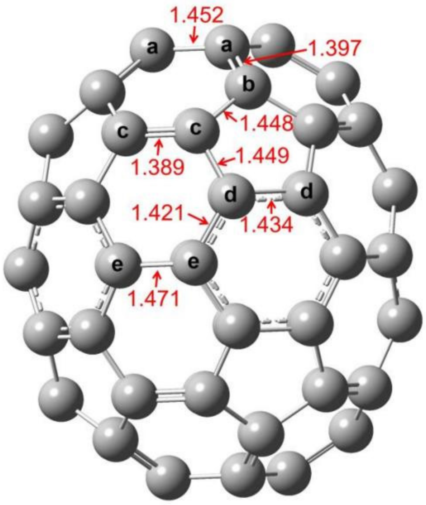

$R_{C\text{-}C}$(Å)	Bonds
< 1.400	**a-b; c-c**
1.400 ~ 1.440	**a-a; b-c; c-d; e-e**
> 1.440	**d-d; d-e**

Figure S8. The optimized structure of D_{5h}-C_{70} at B3LYP/6-31G(d,p) level by G16 program.

Since the final isolated products ($C_{71}H_2$-I, $C_{71}H_2$-II and $C_{71}H_2$-III) were very few, the ^{1}H NMR spectra of these three isomers of $C_{71}H_2$ could not be characterized. Therefore, their ^{1}H NMR spectra were simulated by theoretical calculations.

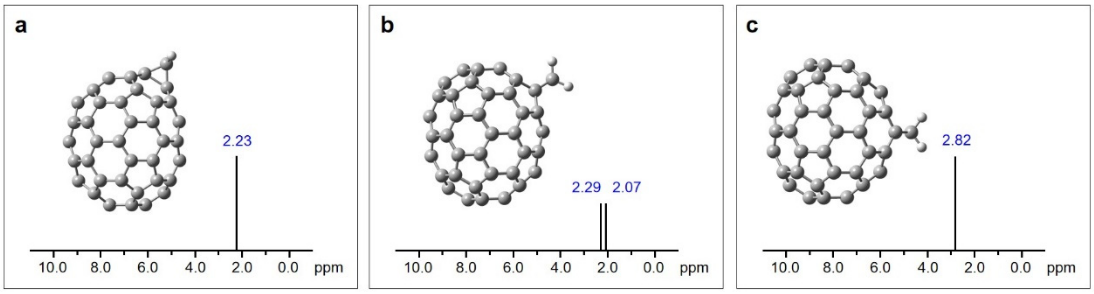

Figure S9. Theoretical simulations for ^{1}H NMR spectra of **a–b** (**a**), **c–c** (**b**) and **e–e** (**c**) at B3LYP/6-31G(d,p) level.
